# Hydroxyurea: Clinical and Hematological Effects in Patients With Sickle Cell Anemia

**DOI:** 10.5539/gjhs.v8n3p252

**Published:** 2015-08-18

**Authors:** Bijan Keikhaei, Homayon Yousefi, Mohammad Bahadoram

**Affiliations:** 1 Health Research Institute, Research Centre of Thalassemia and Hemoglobinopathies, Ahvaz Jundishapur University of Medical Sciences, Ahvaz, Iran; 2 Medical Student Research Committee & Social Determinant of Health Research center, Ahvaz Jundishapur University of Medical Sciences, Ahvaz, Iran

**Keywords:** hydroxyurea, sickle cell anemia, hematological improvement

## Abstract

**Background and Aim:**

It is well known that hydroxyurea impacts on clinical and hematologic indices in sickle cell disease (SCD), we aimed to evaluate the effect of hydroxyurea on clinical and hematological improvement of sickle cell anemia.

**Methods and Materials:**

In this cohort study 48 patients with sickle cell disease were enrolled and pain crisis, severity of pain, acute chest syndrome, the number of hospitalization, the rate of transfusion, spleen size, total Hb, HbF levels, MCV, MCH were compared before and after treatment with HU 10 mg/kg/day/for one year.

**Results:**

In patients with Sickle cell disease Hu significantly decreased the rate of transfusion, hospitalization, spleen size and significantly increased Hb, RBC indices and HbF. Furthermore, we did not find any remarkable adverse effect related to HU during the one year follow up in patients.

**Conclusion:**

We demonstrated that in the course of one year hydroxyurea 10 mg/kg/day can significantly increase HbF, total hemoglobin and RBC indices without any notable side effect in patients with SCD.

## 1. Introduction

Sickle cell disease (SCD) is an autosomal recessive inherited hemoglobinopathy. This condition causes vaso occlusive phenomena and hemolysis due to the substitution of the amino acid valine for glutamic acid at the sixth position on the beta globin chain. As a resulta hemoglobin tetramer (alpha2/beta S2) known as Hemoglobin S (HbS) is produced that is low soluble and polymerized when it is deoxygenated (Inati et al., 2008). SCD occurs worldwide but it is more prevalent in Africans, and its mortality rate in children in developed world is about 0.5-1.0 per 100,000 due to infection, acute chest syndrome and stroke. Regular transfusion is meant to sustain hemoglobin rate above 10. Transfusion helps with ease of movement and slows progressive hyperplasia of bone marrow and hence reduces the risk of heart dialation and face and limb changes due to bone deformation (Bain, 2009; Pack-Mabien & Haynes, 2009; Booth et al., 2010; Keikhaei et al., 2013; Bavarsad Shahripour et al., 2014). The level of Fetal hemoglobin (HbF) in patients with Sickle-cell anemia is different. HbF restricted the intracellular polymerization of sickle hemoglobin, hence, it has a positive impact on SCD (Green and Barral 2011). Recently, it's been demonstrated that some chemical agents such as placental gonadotropin, progesterone, Azacitidine, Milrinone, erythropoietin, arginine butyrate, phenylbutyrate and hydroxyurea rise hemoglobin level with hydroxyurea being the least dangerous of them. Therefore, some drugs such as hydroxyurea (HU) and 5-azacytidinethat motivate HbF formation are practicing treatments for SCD so as to reduce the severity and frequency of SCD episodes (Green & Barral, 2014). Hydroxyurea has a decreasing effect on anemia and reduces the need for HbF due to frequent transfusions. Hydroxyurea is a urea analog which was first synthesized in 1869 by a German chemist. The chief action of hydroxyurea is inhibiting ribonuklexid d-phosphate ridaktaz (RDR) enzyme which partly provides cells with deoxyribonucleotide while copying DNA during cell division. Several studies indicated that 60% of patients with SCD, response to HU treatment, also these studies emphasized 44% of patients experienced reduction of painful episodes; however the mentioned studies detected the occurrence of clinical response after long term treatment with HU (Wong et al., 2014). In addition, a number of studies indicated that hydroxyurea have other mechanisms such as leukocyte count decreasing, red blood cell volume alteration, phosphatidylserine exposure reduction and some other mechanisms that result in several clinical advantages in patients with SCD (Agrawal et al., 2014; Green & Barral, 2014; Wong et al., 2014). In this study we investigated the effect of hydroxyurea on clinical and hematological improvement of sickle cell anemia. We decided to address concerns about safety and effectiveness of HU in patients referred to Shafa hospital, Ahvaz, Iran.

## 2. Methods

In this cohort study, 48 children aged 6-18 years were recruited. All the children had sickle cell disease and were admitted to Shafa Hospital, Ahvaz, Iran, from 2013 to 2014. The criteria for enrollment were sickle cell disease and written consent for participating in the study. Patients were excluded if they had active liver disease, creatinine more than 1.5 mg/dl and treatment other than Hu. The study procedure was explained for all patients and their parents and written informed consent was taken. The study was signed by ethical committee of Ahvaz University of medical sciences and research center for thalassemia and hemoglobinopathy. Specific questionnaires were designed for all children and demographic data such as age and sex and duration of disease were recorded. Moreover clinical manifestations of patients such as pain crisis, severity of pain, chest pain syndrome, the number of hospitalization, number of visits by specialist because of pain and the rate of transfusion were registered. Furthermore, blood test such as CBC, and Hb electrophoresis, HbF measurement, liver and kidney function tests were performed for all patients before treatment and repeated periodically. Then hydroxyurea 10 mg/kg/day was administered for one year. Hydroxyurea dosage was determined according to pediatric section's assessment of endurance and weight of the patient. During this time the patients were referred monthly to Shafa Hospital to receive their medicine. They were watched closely by the pediatric section in case there was a need to repeat tests or take a particular measure. At the end of the study all tests were measured again and any possible adverse effects related to HU were evaluated and recorded. Toxicity for this drug was defined as follows: Neutrophils less than 2000 µl, Platelets less than 80000 µl, Hemoglobin less than 4.5 g/dL, Reticulocyte count less than 0.8%, In case of toxicity with any of the above criteria, the drug was discontinued and after normalization of complete blood cell count, it was continued as 10 mg for every kg of body weight.

Data were analyzed using SPSS version 21. Categorical data were presented as numbers (%), and continuous data as mean ± SD. We used the Chai_2 test to compare categorical variables. α< 0.05 was consider significant.

## 3. Results

In this study 48 patients with SCD were treated with HU and evaluated. These patients consisted of 24 males and 24 females. The minimum age was 6 and the maximum was 18. The initiating age for anemia symptoms for this group of patients was between 2 and 7 years old. The mean initiating age was 13.7 years. No patient had splenectomy. We showed that in patients with Sickle cell disease Hu significantly (P=0.001) decreased the rate of transfusion and 100% of patients became completely transfusion free.

Moreover, HU treatment significantly decreased the rate of hospitalization from 93% to 31.5 %(P=0.03) and number of visits by specialist because of pain reduction from 100% to 6.3% after treatment (P=0.01). The number of pain crisis > 1 decreased from 81.3% to 20.8% (p=0.002). The number of ACS >1 decreased from 81.3% to 20.8 %(p=0.002). Additionally, HU therapy significantly reduced the serum level of MCH<30, Hb F < 20%, MCV<100fl, Hb<10 and significantly increased MCH>30, HbF > 20%, MCV>100fl, Hb>10. (table 1). Regarding response to treatment, in the first 6 months, 60% of patients (24) yeilded more than two fold and in Hbf at end of treatment, this increase was observed in 70% of patients. We showed HU therapy to be well tolerated by our patients and remarkable adverse effects were not reported in patients after one year treatments with HU 10mg/kg/day. Evaluating satisfaction rate of this group of patients, it is revealed that the reason for satisfaction is primarily due to decrease or absence of transfusion and also reduction in bone pain during the course of treatment. Furthermore, reducing fatigue and lethargy was expressed as another reason for satisfaction from patients.

**Table 1:** Clinical manifestations and hematologic indexes in patients with sickle cell disease

Variables		Sickle Cell Disease		P-value	

		Pre Treatment	Post Treatment
Transfusion	yes	32(66.7%)	0	0.001	
	No	16(33.3%)	100	
Hospitalization	No	2(4.2%)	39(68.7)	0.03	
	1	9(18.8%)	10(20.8%)
	2	14(29.2%)	3(6.3%)
	3	19(39.6%)	2(4.2%)
	4	4(8.5%)	0
	total	46(93%)	15(31.3%)
Visit And Hospitalization	no	0	45(93.8%)	0.01	
	1-2	13(27.1%)	1(2.1%)
Because Of Pain	3-4	34(70.8%)	2(4.2%)
	>4	1(2.1%)	0
	total	48(100%)	3(6.3%)
	Crisis	1	9(18%)	17(79.2%)	0.002	
		>1	39(81.3%)	31(20.8%)	
ACS	1	18%	79.2%	0.002	
	>1	81.3%	20.8%	
Hb(G/L)	<6	4(9.1%)	0	0.002	
	6-8	13(27.3%)	9(2.1%)
	8-10	25(52.3%)	28(18.8%)
	10-12	4(9.1%)	10(20.8%)
	>12	1(2.3%)	1(58.3%)
MCH(Fl)	<20	2(4.2%)	8(16.7%)		
	20-27	27(56.3%)	5(10.4%)
	27-30	8(16.7%)	3(6.2%)
	>30	11(22.9%)	32(66.7%)
HbF	<5%	8(16.7%)	0	0.02	
	5-10%	9(18.8%)	0
	10-15%	5(10.4%)	5(10.4%)
	15-20%	6(12.5%)	5(10.4%)
	20-25%	6(12.5%)	14(29.9%)
	>25%	14(29.9%)	24(50.4%)
MCV	<80	28(58.3%)	27(56.3%)	0.2	
	80-100	18(37.5%)	15(31.3%)
	>100	2(4.2%)	6(12.5%)

## 4. Discussion

Previous trial detected that Hydroxyurea is an antimetabolite inhibitor that increases the serum level of total Hb, Hb F, MCH and MCV. Furthermore, it increases transfusion intervals and significantly improves clinical abnormalities (Karimi et al., 2005; Segal et al., 2008). We recruited 48 children with SCD treated with hydroxyurea 10mg/kg/day for one year. We revealed HU significantly decrease the rate of transfusion, hospitalization and number of visits by specialist, moreover improve the level of Hb, MCH, Hb F and MCV. In agreement with our results, Hashemi et al. in a study indicated that Hydroxyurea treatment decreases the numbers of regular transfusion requirement (Hashemi et al., 2009). Moreover, another study in harmony with our findings by Neves et al. showed HU treatment for the duration of one year significantly increases MCV in patients with sickle cell disease (Neves et al., 2012). As mentioned before the valuable effects of HU are fetal hemoglobin induction, decreased cell adhesive properties, inflammation and hypercoagulability (Karimi et al., 2005; Segal et al., 2008). Our results confirmed these findings and showed the level of Hb F improved in our patients after one year of treatment. Consistently, a study by Jeffrey indicated that induction of fetal hemoglobin is an essential mechanism for clinical advantages of hydroxyurea treatmen (Lebensburger et al., 2010). Additionally, another study by Cokicwas in tune with this finding and showed hydroxyurea treatment to increase the level of Hb F in patients with sickle cell disease (Cokic et al., 2003). Other studies also have exposed similar outcomes. A practice by Patel et al. in 2014 supported our results and revealed that treatment with HU at dose 10mg/kg/day significantly increases HbF, total hemoglobin, MCV, and MCH levels (Patel et al., 2014). Furthermore another experience in 2012 by Patel et al. revealed significant increase in serum level of Hb F, total hemoglobin (Hb), MCV and MCH after treatment with hydroxyurea(Patel et al., 2014).

In current trial, the number of pain crisis > 1 decreased from 81.3% to 64.6% after treatment. However the difference was not significant (P=0.08). In contrast Patel et al. in their survey described that HU significantly decreased the rate of pain crisis in patients with SCD (Patel et al., 2014).

The side effects of hydroxyurea are one of the most important concerns. Since some experiments reported several adverse events in patients treated with HU, for example, Ghasemi et al. (2014) indicated dermatologic (39.28%), neurologic (23.2%), gastrointestinal (17.5%) and hematologic (10.71%) events in patients with thalassemia and sickle cell disease. Nonetheless, they highlighted that side effects were transient and non-significant and HU was well tolerated by all patients (Ghasemi et al., 2014). Hence, in current practice we followed up patients both for advantages and possible side effects. All patients in this survey tolerated HU treatment well and did not show any significant adverse effects. Consistently, other studies supported these findings and exposed that HU is a safe agent without remarkable adverse events in children with SCD (Patel et al., 2012)., Also Zamani et al. found no malignancy in five years follow upand only detected one patient with transient thrombocytopenia.

While the carcinogenic effects of long-term HUtherapy is a thoughtful concern no malignancy were found in previous studies regarding HU treatment in patients with sickle cell disease and HU was confirmed to be a safe agent in studies with 5 to 10 yr follow-up (Zamani et al., 2009).

Several limitations are inherent to the present study such as small sample size and short duration of study that limit the ability to generalize the results of our survey. Moreover, this was not a comparative study so we could not compare the effect of HU with other treatment modalities. Further comparative studies are recommended with longer follow-up and larger scales to validate the findings reported here and to answer the question regarding whether HU is a true disease modifiers.

## 5. Conclusion

The results showed hydroxyurea 10 mg/kg/day for one year durationin patients with SCD significantly increased HbF, total hemoglobin, MCV, MCH, and without any remarkable adverse events.

## References

[ref1] Agrawal R. K, Patel R. K, Shah V, Nainiwal L, Trivedi B (2014). Hydroxyurea in sickle cell disease: drug review. Indian J Hematol Blood Transfus.

[ref2] Bain B. J (2009). Neonatal/newborn haemoglobinopathy screening in Europe and Africa. J Clin Pathol.

[ref3] Bavarsad Shahripour R, Mortazavi M. M, Barlinn K, Keikhaei B, Mousakhani H, Azarpazhooh M. R, Alexandrov A. V (2014). Can STOP Trial Velocity Criteria Be Applied to Iranian Children with Sickle Cell Disease?. J Stroke.

[ref4] Booth C, Inusa B, Obaro S. K (2010). Infection in sickle cell disease: A review. Int J Infect Dis.

[ref5] Cokic V. P, Smith R. D, Beleslin-Cokic B. B, Njoroge J. M, Miller J. L, Gladwin M. T, Schechter A. N (2003). Hydroxyurea induces fetal hemoglobin by the nitric oxide-dependent activation of soluble guanylyl cyclase. J Clin Invest.

[ref6] Ghasemi A, Keikhaei B, Ghodsi R (2014). Side effects of hydroxyurea in patients with Thalassemia major and thalassemia intermedia and sickle cell anemia. Iran J Ped Hematol Oncol.

[ref7] Green N. S, Barral S (2011). Genetic modifiers of HbF and response to hydroxyurea in sickle cell disease. Pediatr Blood Cancer.

[ref8] Green N. S, Barral S (2014). Emerging science of hydroxyurea therapy for pediatric sickle cell disease. Pediatr Res.

[ref9] Hashemi A, Abrishamkar M, Jenabzade A. R, Eslami Z (2009). Hydroxyurea can reduce or eliminate transfusion requirements in children with major and intermediate thalassemia. Iranian Journal of Blood Cancer.

[ref10] Inati A, Koussa S, Taher A, Perrine S (2008). Sickle cell disease: New insights into pathophysiology and treatment. Pediatr Ann.

[ref11] Karimi M, Darzi H, Yavarian M (2005). Hematologic and clinical responses of thalassemia intermedia patients to hydroxyurea during 6 years of therapy in Iran. J Pediatr Hematol Oncol.

[ref12] Keikhaei B, Mohseni A. R, Norouzirad R, Alinejadi M, Ghanbari S, Shiravi F, Solgi G (2013). Altered levels of pro-inflammatory cytokines in sickle cell disease patients during vaso-occlusive crises and the steady state condition. Eur Cytokine Netw.

[ref13] Lebensburger J. D, Pestina T. I, Ware R. E, Boyd K. L, Persons D. A (2010). Hydroxyurea therapy requires HbF induction for clinical benefit in a sickle cell mouse model. Haematologica.

[ref14] Neves F, Menezes Neto O. A, Polis L. B, Bassi S. C, Brunetta D. M, Silva-Pinto A. C, Angulo I. L (2012). Hematological differences between patients with different subtypes of sickle cell disease on hydroxyurea treatment. Rev Bras Hematol Hemoter.

[ref15] Pack-Mabien A, Haynes J (2009). A primary care provider's guide to preventive and acute care management of adults and children with sickle cell disease. J Am Acad Nurse Pract.

[ref16] Patel D. K, Mashon R. S, Patel S, Das B. S, Purohit P, Bishwal S. C (2012). Low dose hydroxyurea is effective in reducing the incidence of painful crisis and frequency of blood transfusion in sickle cell anemia patients from eastern India. Hemoglobin.

[ref17] Patel S, Purohit P, Mashon R. S, Dehury S, Meher S, Sahoo S, Patel D. K (2014). The effect of hydroxyurea on compound heterozygotes for sickle cell-hemoglobin D-Punjab--a single centre experience in eastern India. Pediatr Blood Cancer.

[ref18] Segal J. B, Strouse J. J, Beach M. C, Haywood C, Witkop C, Park H, Lanzkron S (2008). Hydroxyurea for the treatment of sickle cell disease. Evid Rep Technol Assess (Full Rep).

[ref19] Wong T. E, Brandow A. M, Lim W, Lottenberg R (2014). Update on the use of hydroxyurea therapy in sickle cell disease. Blood.

[ref20] Zamani F, Shakeri R, Eslami S. M, Razavi S. M, Basi A (2009). Hydroxyurea therapy in 49 patients with major beta-thalassemia. Arch Iran Med.

